# Lessons learned from conducting mental health intervention research in schools in the global south: Our experiences in South Africa and Kenya

**DOI:** 10.1177/13591045231189409

**Published:** 2023-07-13

**Authors:** Maria E Loades, Bronwynè Coetzee, Tom Osborn, Suzanne Human, Katherine Venturo-Conerly

**Affiliations:** 1 Department of Psychology, 1555 University of Bath, Bath, UK; 2 Department of Psychology, 26697 Stellenbosch University, Stellenbosch, South Africa; 3 585906 Shamiri Institute, Nairobi, Kenya; 4 Department of Psychology, 1812 Harvard University, Cambridge, MA, USA

**Keywords:** Adolescent, school-based, mental health interventions, LMICs, Africa

## Abstract

Most of the world’s population of young people live in lower-and middle-income countries (LMICs; (Weine, Horvath Marques, Singh, & Pringle, 2020)), and these young people experience heightened rates of known risk factors for developing mental disorders such as poverty and exposure to trauma (Atwoli, Stein, Koenen, & McLaughlin, 2015). Access to professional psychological treatments is limited in LMICs due to structural barriers (e.g., a dearth of trained professionals) and cultural factors like stigma and beliefs about mental health and illness. Therefore, schools, which are widely attended, may be a good location for providing mental health interventions, and it is important that we develop and evaluate feasible, acceptable, effective, and scalable interventions for use in this context. Yet under 10% of clinical trials of psychotherapies (Venturo-Conerly, Eisenman, Wasil, Singla, & Weisz, 2022) have been conducted in LMICs. And there are particular challenges to conducting research in schools, as has been highlighted in the UK context by Moore et al. (2022). Building on that commentary, our aim herein is to share our learnings from conducting psychotherapy research in schools in Kenya and South Africa.

Most of the world’s population of young people live in lower-and middle-income countries (LMICs; (Weine et al., 2020)), and these young people experience heightened rates of known risk factors for developing mental disorders such as poverty and exposure to trauma (Atwoli et al., 2015). Access to professionally provided psychological treatments is limited in LMICs due to structural barriers (e.g., a dearth of trained professionals) and cultural factors like stigma and beliefs about mental health and illness. Therefore, schools, which are widely attended, may be a good location for providing mental health interventions, and it is important that we develop and evaluate feasible, acceptable, effective, and scalable interventions for use in this context. Yet under 10% of clinical trials of psychotherapies (Venturo-Conerly et al., 2022) have been conducted in LMICs. And there are particular challenges to conducting research in schools, as has been highlighted in the UK context by Moore et al. (2022). Building on that commentary, our aim herein is to share our learnings from conducting psychotherapy research in schools in Kenya and South Africa.

The authors of this paper are from two separate research groups; one group conducted research in South Africa (BC, SH, ML), and the other group did so in Kenya (TO, KVC). These programmes of research were independent of each other. Through a series of conversations, we have shared our learning about conducting psychotherapy research in school contexts in LMICs, and herewith, provide a summary of themes from these discussions. While the examples discussed in this article are specific to the local contexts of our work, they are used to illustrate transferable learnings that may be helpful to others who conduct mental health research in school settings, both in and beyond LMICs. We first introduce our respective programmes of work to set the context, following which we present the learning points, drawing on our own examples.

## Introducing Our Work

### South Africa: Developing 4 Steps To My Future in Primary Schools

In South Africa, there is a high level of mental health need, and a lack of access to help. Of the 18.5 million children and young people (CYP) in South Africa, 4.8 million do not live with either parent (StatsSA, 2013), 42% have experienced maltreatment, and 82% have experienced or witnessed victimisation, 33% have experienced physical or sexual abuse, 25% have experienced emotional abuse, and 20% have experienced neglect (Artz et al., 2018). These contextual factors place South African young people at considerable risk for mental health problems. There are no national prevalence data on common mental health problems in South Africa, but estimates suggest that around 15% of children (age 10 – 12) have anxiety or depression (Cortina et al., 2016) and 40% of young people (age 14 – 15) could have depression (Das-Munshi et al., 2016). Despite this considerable mental health burden, access to help is limited by the lack of formally trained providers (Lund et al., 2011; Patel et al., 2010). Indeed, available data suggest that per 100 000 people, South Africa has 1.52 psychiatrists, substantially lower than the median number of 2.1/100 000 for upper-middle income countries, and 12.7/100 000 in high income countries (W.H.O., 2018). Most CYP attend school, creating a potential site for providing accessible mental health support. Our research in the Western Cape at the southern tip of South Africa took place in collaboration with Community Keepers (CK), a local, school-based non-governmental organisation (NGO) which provides psychosocial support (https://communitykeepers.org/).

Mental health interventions are more likely to be successfully implemented at schools that partner with mental health organisations (Langley et al., 2010) and consistent with this, CK currently works in 35 public primary and secondary schools, and aims to be in 100 schools by 2030. CK partners with each school to provide individual and/or group psychotherapeutic support to CYP with symptoms of mental health problems, as well as more proactive, psycho-educational skills workshops to CYP, parents and teachers. CK is staffed by psychologists, registered counsellors and social workers. The problem we identified and secured Wellcome Trust funding to address was the lack of culturally and contextually appropriate proactive interventions to be delivered at classroom level to young adolescents, who are disproportionately vulnerable to developing mental health problems as they transition to high school and enter puberty.

We developed *4 Steps To My Future (4STMF)*, a Cognitive Behavioural Therapy (CBT)-informed, psycho-educational intervention for children in grades 5–7 (approximately age 10 – 15 years of age) based on the following: (1) individual, semi-structured, in-person interviews with 66 children and young people, parents (primary caregivers), teachers, and CK staff members (Coetzee et al., 2022b), (2) a systematic review of universal school-based mental health programmes delivered in LMICs (Bradshaw et al., 2021), and (3) local and international experts in research, CBT, and intervention development and evaluation with CYP. In our interviews with key stakeholders, participants spoke about the need for the programme to be able to fit in with school schedule and for us this meant being able to deliver a structured, and somewhat brief programme with a clear beginning and end (Coetzee et al., 2022b). Our systematic review (Coetzee et al., 2022b) found that no universal interventions had previously been delivered in the South African context. Further, 3 out of the 12 universal interventions delivered in LMICs were supported by CBT as an underlying theory. Our rationale for developing an intervention underscored by the principles of CBT was motivated by the evidence-base for the effects of CBT on depression and anxiety (Hayes et al., 2023). Through a series of meetings, we developed an initial prototype of the programme, drawing on the expertise within the group, with Prof Paul Stallard having designed, developed, piloted and tested several universal-school based interventions (informed by CBT) in the UK, Dr Maria Loades is a CBT practitioner, supervisor and trainer in the UK with expertise in working with adolescents with depression, and Prof Helene Loxton having developed CBT based interventions in the South African school context and taught South African clinical trainees. We then spent 2 days working through the prototype manual, role playing it session by session with staff from our partner organisation, Community Keepers, and refined it considerably based on their input and context-specific knowledge.

In essence, 4STMF is designed to be universally delivered to all children at classroom level, the four core steps of *4STMF* focus on enhancing self-esteem, promoting helpful thinking, developing emotional regulation skills, and empowering goal-focused action and problem-solving. The programme comprises 8 sessions, each about 25 minutes in length. We conducted a feasibility trial in two urban, public, primary schools within which CK operates, both of which are part of South Africa’s National School Nutrition Programme, indicating that most learners at these schools come from low socio-economic status (SES) families. We delivered the intervention to all grade 5 children (age 10–13 years) via trained post graduate psychology students (Coetzee et al., 2022), finding indications of feasibility and acceptability, even within the COVID-19 pandemic context (Coetzee, Loades, Human, Gericke, Laning, Kidd, Stallard, *under review*). Across all grade 5 learners in two schools, only two parents returned parental opt-out consent forms indicating that they did not want their child to participate in the study, and most eligible learners at both schools agreed to participate (85% - school 1; 91% - school 2). All learners completed baseline measures, and more than 80% completed post-intervention measures. Learner session attendance and programme fidelity were high. Feedback from teachers (a study-specific feedback form) and learners (focus groups) indicated that the learners enjoyed the programme, and were able to share the content they had remembered with family at home.

### Kenya: Implementing Shamiri for High School Students

The Kenyan population is rather youthful with a median age of only 19, and approximately 15% of the Kenyan population are aged 13 to19. Approximately 80% of them attend secondary (high) schools. Sadly, recent studies estimate that about half of school-going Kenyan adolescents have elevated symptoms of common mental health problems (Osborn, Venturo-Conerly, et al., 2019). And barriers similar to those in South Africa inhibit help-seeking for youth mental disorders, including a paucity of professional caregivers (W.H.O., 2019) the length and cost of traditional psychotherapy in a country where incomes are low, and continued government under-investment in mental healthcare (Patel et al., 2007). Thus, the urgent need for mental health interventions may be met, at least in part, through accessible school-based interventions.

Our intervention development efforts in Kenya have been guided by three important ideas: (1) community-based delivery of mental healthcare increases access, reduces stigma, and enhances community involvement (Wasil et al., 2021), (2) task-shifting to lay-providers can be an effective way of expanding the caregiving workforce (Venturo-Conerly et al., 2022) and (3) brief and simple interventions that focus on broad, universal, and often positive psychological principles—rather than an explicit reference to the treatment of psychopathology—can be effective for common mental health problems (Walton, 2014).

Combining these three ideas, we developed the “Shamiri” intervention. Shamiri is a Kiswahili word for “thrive”. The Shamiri intervention combines three evidence-based brief interventions into a single treatment protocol. These three elements are *growth-mindset,* which teaches young people that personal characteristics and attributes are malleable; *gratitude*, which teaches them to notice and appreciate the good things that happen to them; and *value affirmation*, which encourages them to reflect on self-defining values and take value-aligned actions. The intervention is delivered by near-peer lay-providers, aged 18 to 22 years, after only 10 hours of training. This is a group–based intervention, delivered in schools. Students are gathered in groups of 6 to 15 and complete four weekly one-hour group sessions: the first session is focused on neuroplasticity and the human capacity for growth, the second on strategies for growth, the third on noticing an expressing gratitude, and the fourth on identifying personal values and related goals. Importantly, the Shamiri intervention was developed through multi-cultural and inter-disciplinary collaboration. The research team consisted of researchers and practitioners in Kenya, U.S., and Europe as well as local teachers and educators, entrepreneurs, and young people with lived experiences.

We tested the Shamiri intervention across four completed and several ongoing randomised controlled trials (RCTs), all conducted in Kenyan high schools. Information about Shamiri lay-provider training and supervision can be found in Venturo-Conerly et al. (2022). In a pilot RCT with 51 young people (aged 14–17) with elevated levels of depression (Patient Health Questionnaire-8) or anxiety (Generalized Anxiety Disorder Screener-7) symptoms, significant reductions in depression and anxiety as well as improvement in academic performance and social support from baseline to the 4-week endpoint were observed in the intervention group relative to the active control (Osborn et al., 2019). A well-powered replication RCT with 413 young people (aged 13–18) with elevated symptoms of anxiety (GAD-7) or depression (PHQ-8) found significant reductions in depression and anxiety symptoms (Osborn et al., 2021) again at 4-week endpoint; importantly, we found that these effects were maintained across the 7-month follow-up (Osborn et al., 2021).

## What We Have Learned

### Keep the End Goal of Implementation at Scale in Mind From the Outset

Key to the 4STMF pilot study was to be able to design and develop this universal intervention (the first of its kind in SA) in partnership with schools. This partnership meant that we could understand the barriers and facilitators to participation though our formative interviews pre-COVID and during COVID with key informants (Coetzee et al., 2022a; Coetzee et al., 2022b), and therefore determine at an early stage whether the programme could be potentially integrated into the existing curriculum. A key learning point for us was that sessions had to be no more than 25 minutes in length to fit into existing timetables, and to be offered in both English and Afrikaans, the two local languages spoken in both schools. Schools offered space within the Life Orientation (LO) lessons to deliver our sessions. As LO lessons are designed to provide learners with knowledge and skills about personal and social well-being, health education, social and environmental responsibility, career development and study skills as well as relationships and sexual education, a programme like 4STMF could sustainably be integrated into the LO curriculum at scale in the future. Although we had hoped to offer workshops to parents and caregivers in parallel to the classroom sessions for the learners, our formative work highlighted barriers to parents being able to take up such an offer, given their work commitments, and we therefore developed brief leaflets to send home via the learners as a way to share key points from 4STMF with parents.

Working in partnership with organisations who are already providing psychosocial support in the community is also key to implementation and sustainability. From the outset, including Community Keepers staff members as official co-investigators on the 4STMF study also meant that we set out with sustainability in mind, and remained focused on questions like how and by whom might the intervention be delivered in the future. While our interventionists were psychology graduates, future delivery might be possible through CK staff – which includes a cadre of young people trained as care facilitators through CK. At the time of this writing CK has already expanded to more than 35 schools in South Africa, with considerable investment to add an additional 60 by the end of 2023. As such, there is much benefit for schools in partnering with NGO’s like CK to provide psychological support to learners.

### Silos and the Incentive Problem: What’s in it for All of Us?

When researchers conceptualize and design studies, it is natural to think about study design from a researcher’s perspective. For example, researchers are likely to focus on important research issues such as hypotheses and study questions, measures, study design, and statistical power and confounding variables. And most often, we think of schools, and by extension school staff, primarily as “study sites” rather than as members of the research team. Therein lies two problems: first, an all too common problem is that this can cause studies to be designed in silos (Collyer & Smith, 2020; Mead et al., 2021), and second, we are unlikely to think about the following important question: *what is in it for schools and school staff?*

We have, in our experience, considered these two problems (the “silos” and “incentives” problems) and how to address them in the context of our work in schools. Re-aligning and merging incentives and embracing co-creation and collaboration from the onset. Let us first talk about re-aligning and merging incentives. It may seem that researchers and schools have different incentives, but upon close inspection, their incentives often align. In our work in Kenya, for example, all parties (I.e., the researchers, school staff, teachers, parents, and even students) shared a common goal: for students to thrive and succeed. We therefore framed our work to school staff, teachers, parents, and students, as research that could help students thrive and succeed. Finding that common incentive across all the stakeholders and framing the research around that shared incentive is a powerful way of creating buy-in.

Similarly, meaningful co-creation and collaboration can be very powerful in getting buy-in from school staff and other key community organisations. Crucially, co-creation should be meaningful if it is to produce buy-in. For example, seeking input from school staff after a full study has already been designed is not meaningful co-creation but more akin to rubberstamping. School staff, so long as they have capacity and interest, should be involved from much earlier in the process, as this may allow them to feel a sense of ownership over the study and therefore support its implementation. And when co-creating with school staff, it is important to provide due credit, for example, by making an important member of the school staff a co-author on manuscripts or finding other ways of acknowledging and sharing our success with them. Another example, pivotal to building our partnership with Community Keepers in South Africa was a 2-day intensive ‘training’ on the intervention manual. Our plan was for the intervention to be facilitated in the pilot trial by our research team; however, we extended the invite to the facilitator training to all CK staff working in primary schools at the time (*N* = 20), all of whom attended. Rather than approaching the training didactically, ML led the training using role play to interactively work through the manual and intervention materials, inviting and actively welcoming reflections and suggestions about how to improve these to better fit the context, based on CK’s expert knowledge and experience on the ground. This was mutually beneficial; it resulted in a much more ‘implementable’ intervention, fit for context and culturally sensitive, and empowered the CK partners through valuing their expertise and sharing evidence-based ideas with them that they could use in their therapeutic practice. It also dismantled the silos. 

### “Things are Different on the Ground”, Balancing Fidelity and Realities of Implementation

“Things are different on the ground,” is a common saying in Kenya. The idea that well laid out plans must still adapt to the realities of implementation is highly relevant to school-based interventions. From macro–level factors like changes to school staff and calendars to micro–level factors like some students missing due to unpaid school fees or competing activities, it is nearly impossible to plan for all the factors that may influence study protocol implementation in schools. Therefore, study protocols should be designed to have “breathing room” to adapt to the constantly changing school environment. As an example of the flexibility needed for implementation, several of the authors were to commence baseline assessments in two primary schools in South Africa the same week that COVID-19 restrictions were imposed in March 2020, and schools shut down. This meant delaying the planned pilot trial by a full year, commencing in 2021, and changing some intended delivery plans to fit with ongoing social distancing and disease containment measures and policies at that time, for example the wearing of face masks to cover an individual’s mouth and nose, and sanitizing hands upon entering and leaving a classroom. Initially the 4STMF programme included some small group activities that children would have been encouraged to do together, but these small group activities were replaced by individual activities, with each child having their own individual worksheet to complete at their own desk. We retained interactive activities where these could be done within what was permitted for social distancing, for example, an activity where children worked in pairs which could be implemented with children remaining seated at their respective desks and talking to the child seated next to them without getting up out of their seats. Thus, while some physical aspects of the delivery of 4STMF had to be changed, the content remained the same. Although the global pandemic is a more extreme example of the need to be flexible and adaptable in the face of uncertainty and change, this theme resonates throughout our work in schools - expect the unexpected.

### Measuring What Matters and Measuring it Meaningfully

Imagine telling a high-school principal in rural Kenya that if an intervention works, their students will experience a significant reduction in mental health problems measured by the Patient Health Questionnaire (PHQ-9) with an effect size of Cohen’s d = .4. This most likely would just confuse the principal, as these terms are only familiar to researchers and clinicians (Hanel & Mehler, 2019). In our work in Kenya, we use the PHQ to measure depression symptoms. Unfortunately, outside of clinicians and researchers, the average person doesn’t really understand what a two – point reduction in PHQ scores really means and whether it makes a meaningful difference in their lives. Therefore, sharing findings in ways that are more intuitive to the non-scientist (e.g., simple percentages and visual representations of statistics, (Franconeri et al., 2021)) may improve communication with schools. Additionally, the outcomes that matter most to school staff may not be clinical, but instead focused on constructs like academic performance, social relationships within the school, and school climate (Krause et al., 2021). In our work in Kenya, we decided to include these important outcomes as secondary measures in our study design and to share these outcomes with the school staff. In our work in South Africa, we had research assistants attend all 4 Steps To My Future sessions and rate how much the CYP engaged with, participated in, enjoyed, and understood the various activities. Teachers were also invited to provide feedback on what they thought of the facilitators and the programme, as well as to reflect on how the implementation of the programme may have influenced their work responsibilities at school. It is important to obtain feedback such as this from multiple stakeholders and to incorporate the feedback in future research, especially when thinking about the scaling up of such mental health interventions at more schools. Overall, measuring what matters involves consideration for the measures and outcomes that matter to all stakeholders, including the school staff, and communicating those findings as appropriate.

An ongoing problem for researchers is that many of the existing mental health and functioning measures, including those now strongly encouraged by international funding agencies, are built and tested in Western countries. As such, our understanding of the constructs measured by these Western-derived instruments rely on assumptions about the generalizability of Western-derived research methods and nosological constructs across diverse populations (Atilola, 2015). And whereas there is a lot of utility in using similar measures across cultures, to develop a robust understanding of what we are measuring, we should aim to complement these tools with culturally adapted or locally co-developed measures (Nyongesa et al., 2022; Tele et al., 2023). Thus, we need to measure what matters for all stakeholders and school staff (and through co-creation we can identify what matters to different stakeholders) and we need to measure it meaningful by taking into consideration not only psychometric properties but whether the measure captures all culturally – salient features of the outcome we are assessing.

## Conclusion

Through describing our work and our learning, we have summarised some of the key issues school mental health intervention researchers working in LMICs may wish to consider. Beyond this, we conclude that we all have a moral and ethical imperative to play the long-game: we need to focus on developing structures and interventions that are sustainable beyond the period of research funding. To do this, we must consider implementation and scalability at every stage of our research activities, including when designing intervention programs and testing intervention outcomes. Ideally, intervention programmes will be designed and tested with real-world implementation in mind, and intervention outcomes may be used to communicate with stakeholders involved in implementation and to inform further collaboration and scale-up involving stakeholders beyond the research team.
